# Supplementary material for: Atypical rhizobia trigger nodulation and pathogenesis on the same legume hosts
Source: Nat Commun. 2024 Oct 26;15:9246. doi: 10.1038/s41467-024-53388-x (PMC11513132; doi:10.1038/s41467-024-53388-x)
Supplement: Supplementary file 1 — Supplementary Information [file 41467_2024_53388_MOESM1_ESM.pdf]

Atypical rhizobia trigger nodulation and pathogenesis on the same legume hosts

This PDF document includes:

Supplementary Figures 1 to 21
Supplementary Methods
Supplementary References

Supplementary Figures

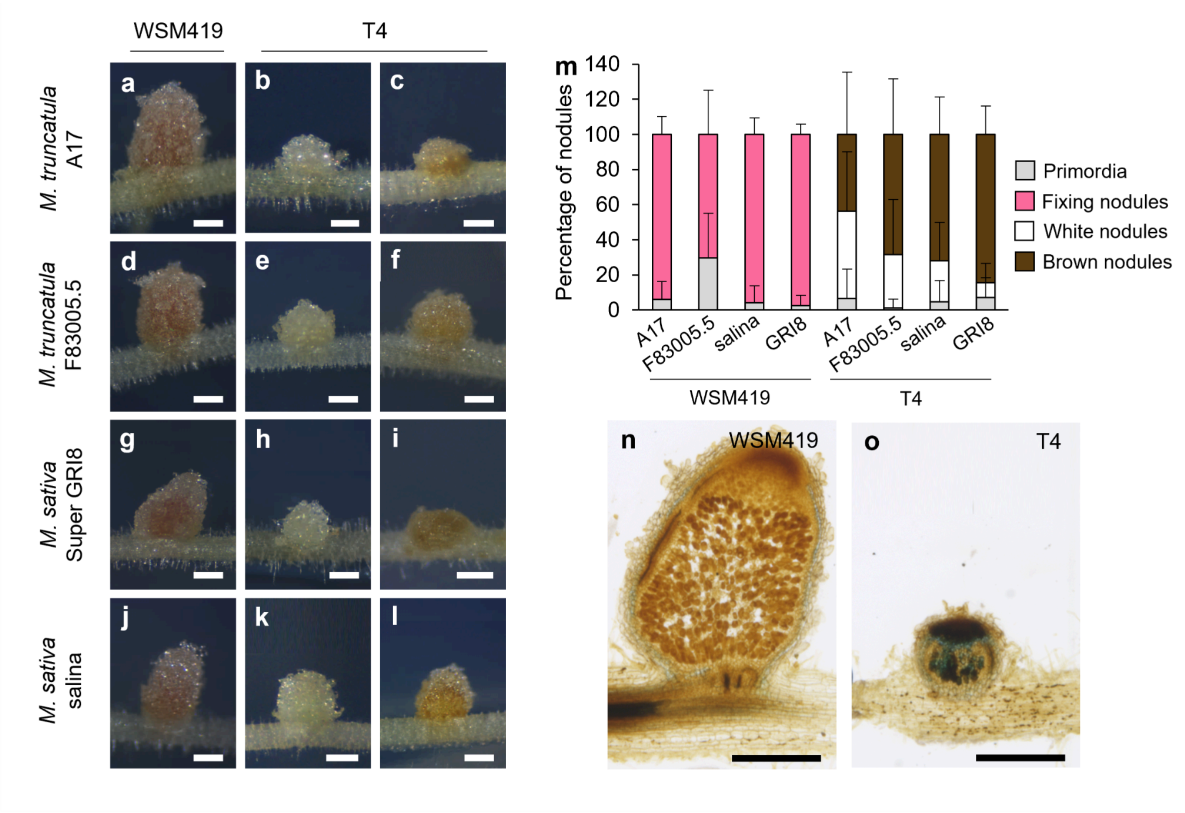

Supplementary Fig. 1 | Phenotypes of nodules induced by T4 on *M. truncatula* and *M. sativa*, and phenolic accumulation. a-l Phenotypes of 14-dpi WSM419- and T4-derived nodules on two genotypes of *M. truncatula* (A17 and F83005.5) and two genotypes of *M. sativa* (Super GRI8 and salina). **a, d, g, j** Nitrogen fixing pink nodules induced by WSM419. **b, c, e, f, h, i, k, l** Non-fixing white-to-brown, small nodules induced by T4. (Scale bar **a-l**, 500 µm). **m** Percentage of the different types of nodules present on *M. truncatula* (A17 and F83005.5) and *M. sativa* (Super GRI8 and salina) when inoculated by WSM419 or T4 (n = 24). **n** and **o** Methylene blue staining of 21-dpi *M. truncatula* A17 nodules inoculated with WSM419 or T4. **n** WSM419 nodules do not accumulate phenolics. **o** T4 nodules accumulate phenolics (blue coloration). (Scale bars **n** and **o**, 500 µm).

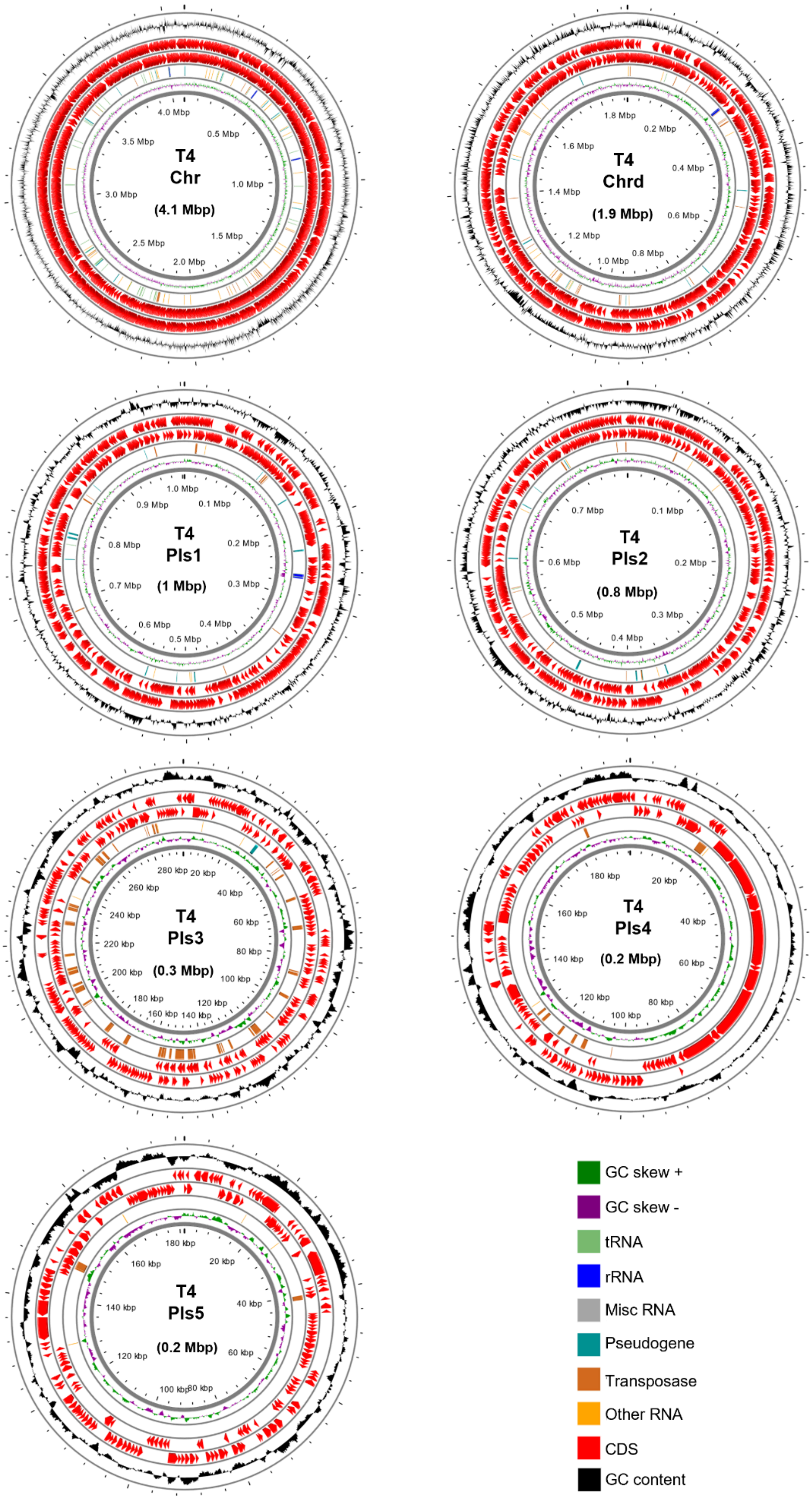

Supplementary Fig. 2 | Maps of T4 replicons. Overview of the seven circular replicons of T4. T4 possesses one chromosome (Chr), one chromid (Chrd) as well as five plasmids (Pls1, Pls2, Pls3, Pls4 and Pls5). GC skew, tRNA, rRNA, misc RNA, pseudogene, transposase, other RNA, CDS and GC content are shown.

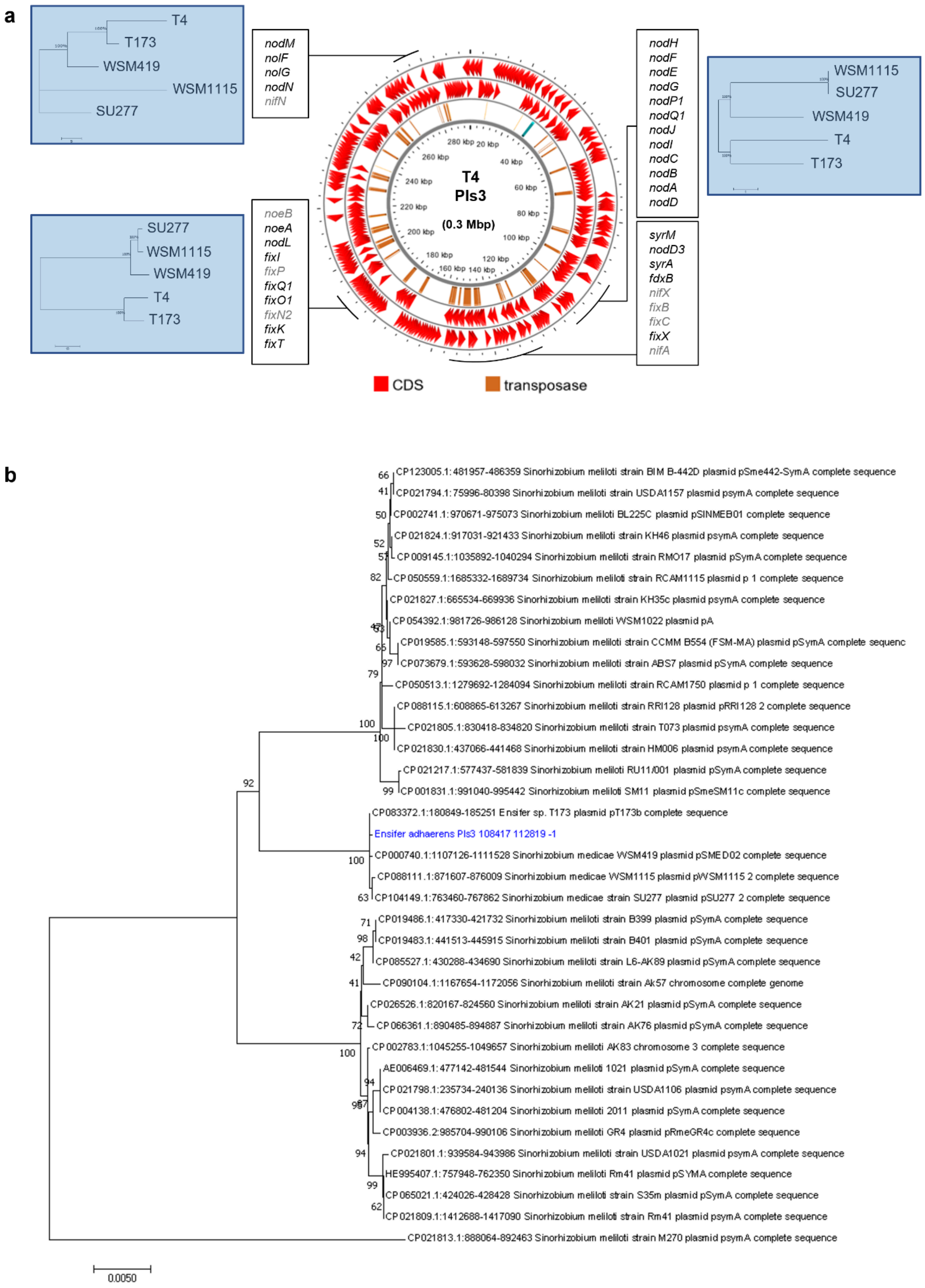

Supplementary Fig. 3 | Symbiotic clusters of the T4 plasmid three and Neighbor-Joining phylogenetic reconstruction of the *nodABCIJ* genes. a Circular view of the T4 plasmid three (T4 Pls3) carrying the nodulation genes and the vestigial nitrogen fixation genes partitioned onto four distinct loci. The density of

transposases is remarkably high in T4 Pls3. Repeated regions in T4 Pls3 (27%) represent more than ten times what is observed in other replicons of T4 (< 3%). Black and grey genes in boxes correspond to presumed-functional genes and pseudogenes, respectively. For three of the four symbiotic gene clusters identified, Maximum Parsimony phylogenetic trees indicate that T4 symbiotic loci are closely related to T173 and secondly to WSM419. **b** Phylogenetic reconstruction of the *nodABCIJ* genes. The evolutionary history was inferred using the Neighbor-Joining method[1]. The optimal tree with the sum of branch length = 0.09774791 is shown. The percentage of replicate trees in which the associated taxa clustered together in the bootstrap test (1000 replicates) are shown next to the branches[2]. The tree is drawn to scale, with branch lengths in the same units as those of the evolutionary distances used to infer the phylogenetic tree. The evolutionary distances were computed using the Maximum Composite Likelihood method[3] and are in the units of the number of base substitutions per site. The analysis involved 37 nucleotide sequences. Codon positions included were 1st+2nd+3rd+Noncoding. All ambiguous positions were removed for each sequence pair. There were a total of 4414 positions in the final dataset. Evolutionary analyses were conducted in MEGA7[4].

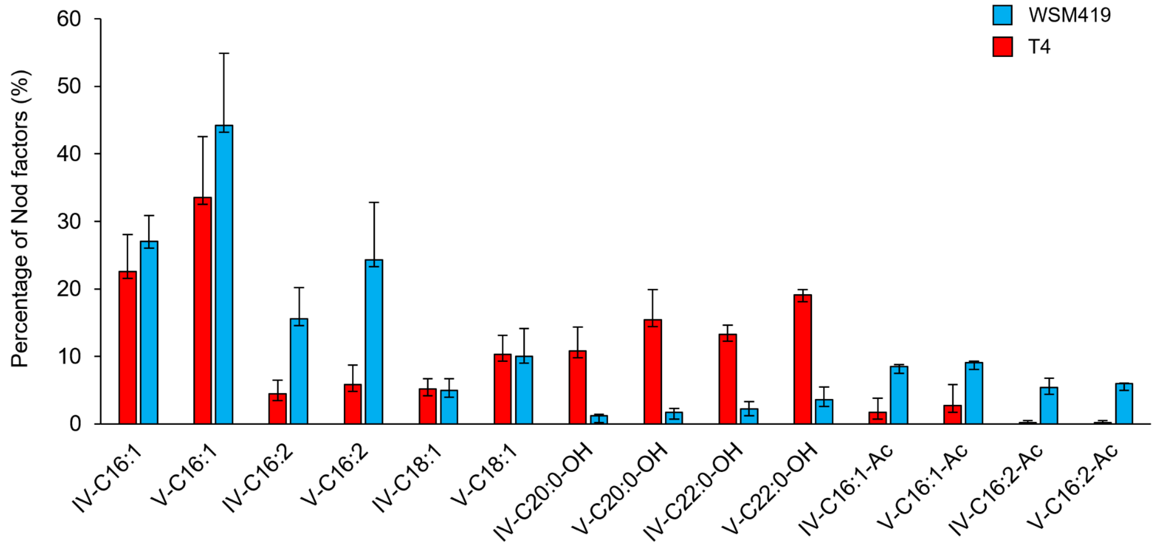

Supplementary Fig. 4 | Main sulfated Nod factors produced by T4 and WSM419. Percentage of sulfated Nod factors produced by T4 (red bars) and WSM419 (blue bars). IV and V, four and five N-acetyl-D-glucosamine (GlcNAc) residues respectively; C, acyl chain [saturated (0) and hydroxylated (OH) or unsaturated (1, 2)]; Ac, acetylated. Three independent experiments were done.

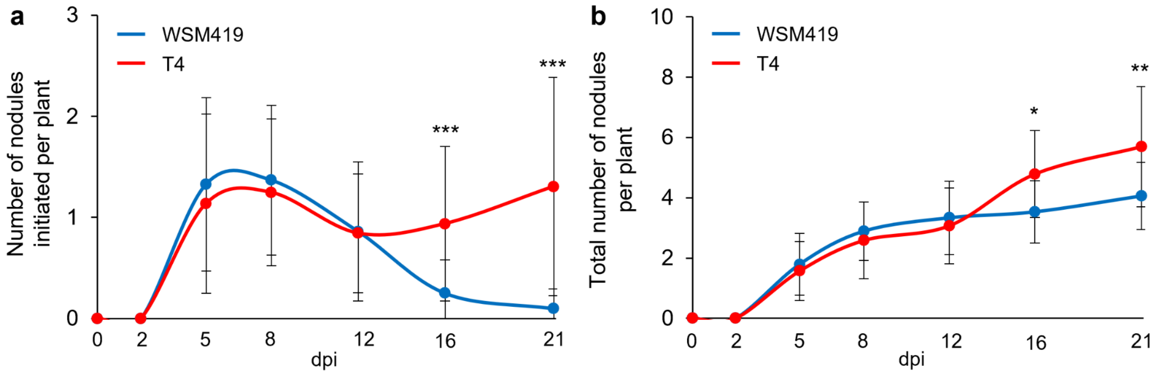

Supplementary Fig. 5 | Nodulation kinetics of T4 compared to WSM419. a and **b** Nodulation kinetics of WSM419 (blue curves) and T4 (red curves). **a** Nodule initiation kinetics. **b** Nodule accumulation kinetics. Results represent means ± SEM of three biological replicates (For 0, 2, 5, 8, 12, 16 and 21 dpi n = 360, 360, 360, 288, 216, 144 and 72 plants, respectively). Asterisks indicate significant differences (*, $P \leq 0.01$; **, $P \leq 0.001$; ***, $P \leq 0.0001$; Student's *t*-test).

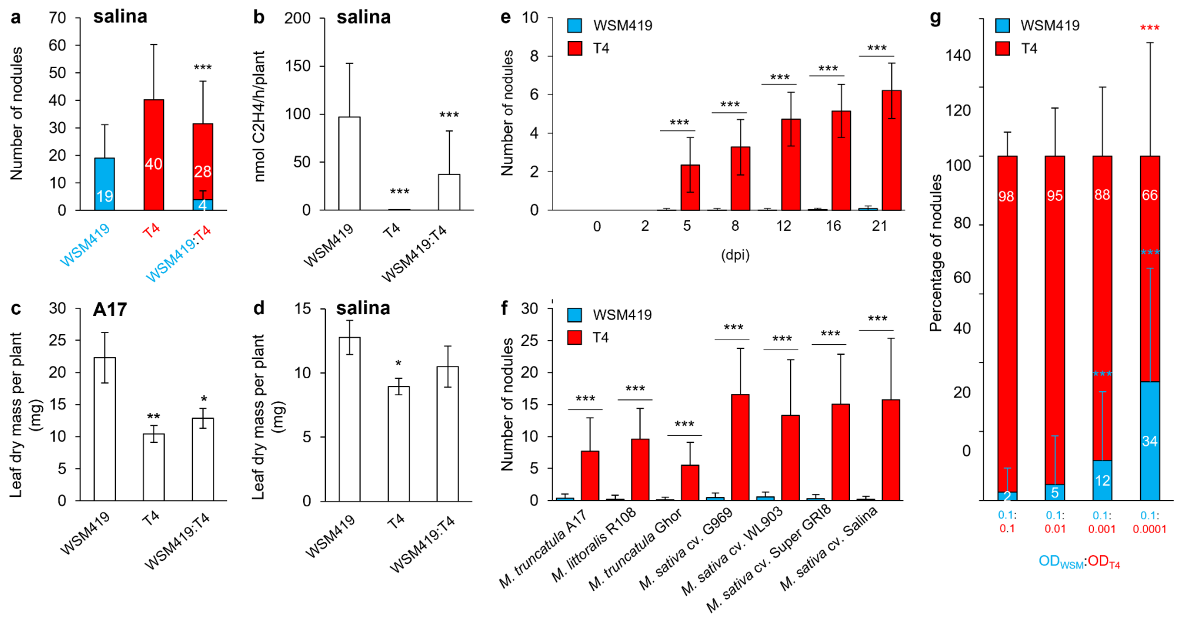

Supplementary Fig. 6 | T4 competitiveness. a-b WSM419 and T4 single and co-inoculation on *M. sativa* cv. *salina*. **a** T4 induced more nodules than WSM419 upon single inoculation and reduced the formation of WSM419 nodules when co-inoculated. Data represent means ± SD of three biological replicates (n = 30 plants). **b** T4 did not fix nitrogen and reduced nitrogen fixation of plants when co-inoculated with WSM419. Data represent means ± SD of three biological replicates (n = 30 plants). **c** and **d** *M. truncatula* A17 **(c)** and *M. sativa* cv. salina **(d)** plants inoculated with T4 or co-inoculated with WSM419:T4 presented a reduced leaf dry mass. Data represent means ± SD of three biological replicates (n = 3). **e** T4 was more competitive than WSM419 for nodule formation during nodulation with *M. truncatula* A17. Data represent means ± SD (n = 160 plants). **f** T4 was more competitive than WSM419 for nodule formation on both *M. truncatula* and *M. sativa* independently of the plant genotype. Data represent means ± SD (n = 40 plants). **g** Under WSM419:T4 co-inoculation, reducing the concentration of T4 allowed to increase the formation of WSM419 nodules. Data represent means ± SD of two biological replicates (n = 80). **a-g** Plants were analyzed at 21 dpi. Asterisks indicate significant differences (*, $P \leq 0.05$; **, $P \leq 0.01$; ***, $P \leq 0.001$; Student's *t*-test).

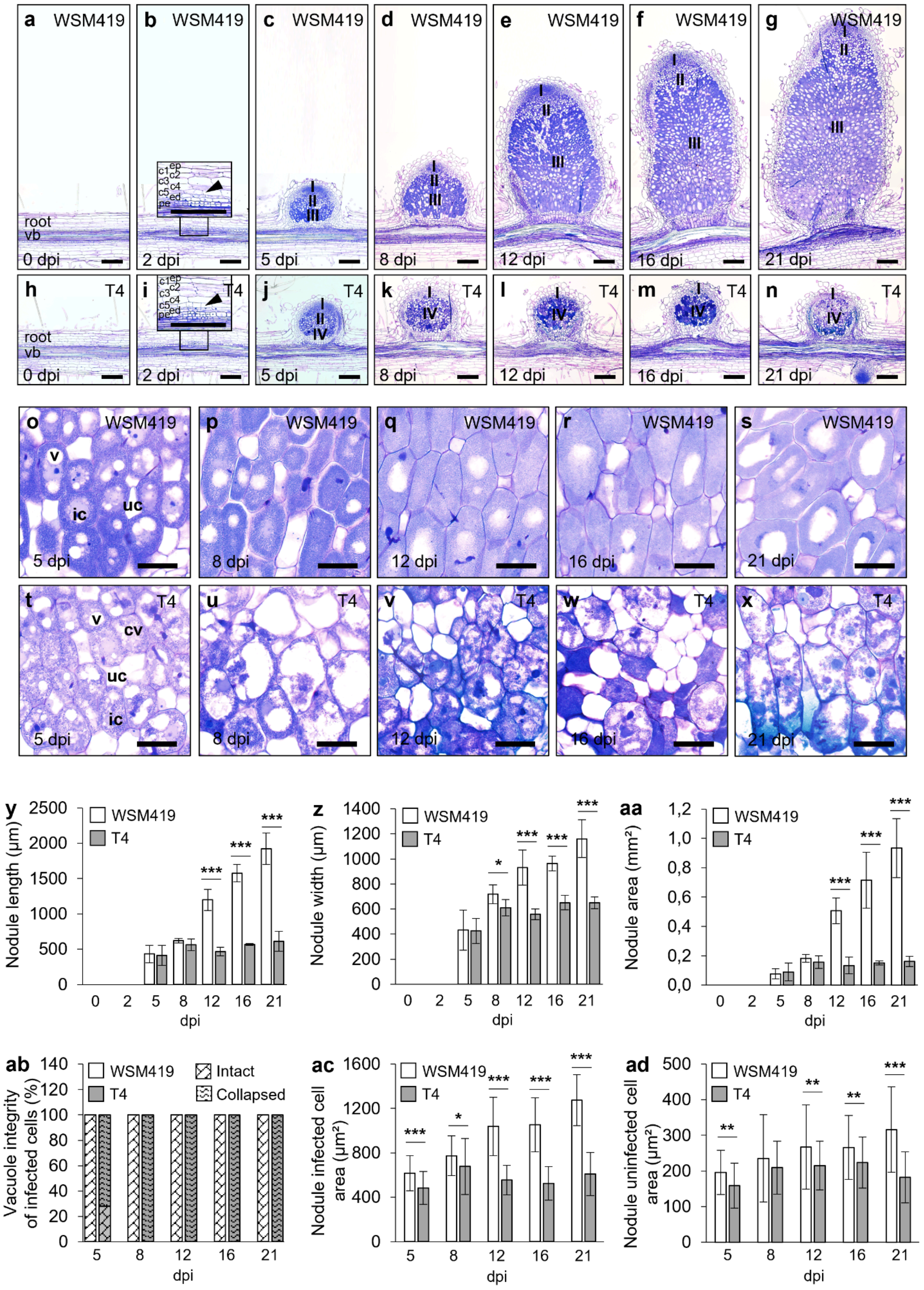

Supplementary Fig. 7 | Quantitative analysis of the T4 nodule histological features. a-n Time series histological analysis of WSM419 **(a-g)** and T4 **(h-n)** nodule development at 0, 2, 5, 8, 12, 16 and 21 dpi. **a** and **h** Primary roots at zero dpi. **b** and **i** Primary roots at two dpi showing nodule organogenesis. Inlets in **(b)** and **(i)** show magnification focusing on cell divisions occurring in pericycle, endodermis and

occasionally in the fifth cortex cell layer (black arrowheads), corresponding to nodule organogenesis stage I[5]. **c-g** WSM419 nodules elongate and show a typical indeterminate nodule structure with a meristematic zone (I), an infection zone (II) and a fixation zone (III). **j-n** T4 nodules do not elongate and do not show a typical indeterminate nodule organization. **j** T4 nodule holds a meristematic zone, an infection zone, lacks the fixation zone and shows a premature senescence zone (IV). **k-n** T4 nodules only present an inactive meristematic zone and a senescence zone. vb, vascular bundle; ep, epidermis; c1, first cortex cell layer; c2, second cortex cell layer; c3, third cortex cell layer; c4, fourth cortex cell layer; c5, fifth cortex cell layer; ed, endodermis; pe, pericycle; I, nodule meristematic zone; II, nodule infection zone; III, nodule fixation zone; IV, nodule senescence zone. (Scale bars **a-n**, 200 µm). **o-x** Time series histological analysis of WSM419 **(o-s)** and T4 **(t-x)** nodule infected cell development at 5, 8, 12, 16 and 21 dpi. **o-s** WSM419 healthy nodule infected cells densely filled with differentiated bacteroids. **t-x** T4 unhealthy nodule infected cells poorly filled with undifferentiated bacteroids showing senescence features. **t** 5-dpi nodule showing a mixture of infected cells carrying intact and collapsed vacuole. **u-x** From 8 to 21 dpi, nodule infected cells present strong senescence features with only collapsed vacuoles. v, vacuole; cv, collapsed vacuole; uc, uninfected cell; ic, infected cell. (Scale bars **o-x**, 40 µm). **y-aa** Measurements of WSM419 (white bars) and T4 (grey bars) nodule length (**y**), width (**z**) and area (**aa**) at 0, 2, 5, 8, 12, 16 and 21 dpi. From 5 to 21 dpi, the length, the width and the area of WSM419 nodules increase while it doesn't in T4 nodules. Asterisks indicate significant differences compared to WSM419 (*, $P \leq 0.01$; **, $P \leq 0.001$; ***, $P \leq 0.0001$; Student's *t*-test). **ab-ad** Characterization and measurement of WSM419 (white bars) and T4 (grey bars) nodule infected and uninfected cells at 5, 8, 12, 16 and 21 dpi. **ab** WSM419 nodules always show 100% of intact vacuoles (diagonal bricks). At 5 dpi, T4 nodules show 70 % of collapsed vacuole (waves) and from 8 to 21 dpi, T4 nodules show 100% of collapsed vacuole. (For each time point and each condition, n = 50 infected cells). **ac** and **ad** From 5 to 21 dpi, the area of WSM419 nodule infected cells (**ac**) and uninfected cells (**ad**) increases while it doesn't in T4 nodule infected and uninfected cells. Asterisks indicate significant differences compared to WSM419 (*, $P \leq 0.05$; **, $P \leq 0.01$; ***, $P \leq 0.001$; Student's *t*-test). (For each time point and each condition, n = 50 infected cells and 50 uninfected cells).

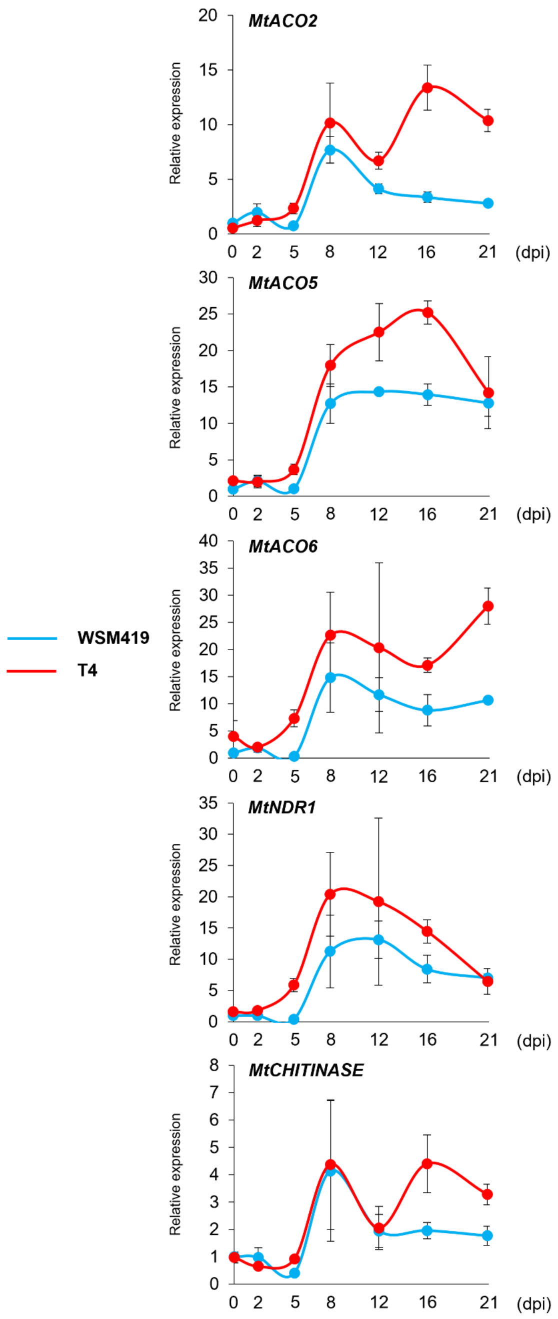

Supplementary Fig. 8 | Gene expression profiling of ethylene biosynthesis and defense marker genes. RT-qPCR gene expression profiles of the putative ethylene biosynthesis marker genes *MtACO2*, *MtACO5* and *MtACO6,* and of the defense marker genes *MtNDR1* and *MtCHITINASE* along WSM419 (blue curves) and T4 (red curves) nodulation kinetics. Data represent means ± SEM of three biological replicates. Gene accessions of amplified genes are provided in **Supplementary Data 4**.

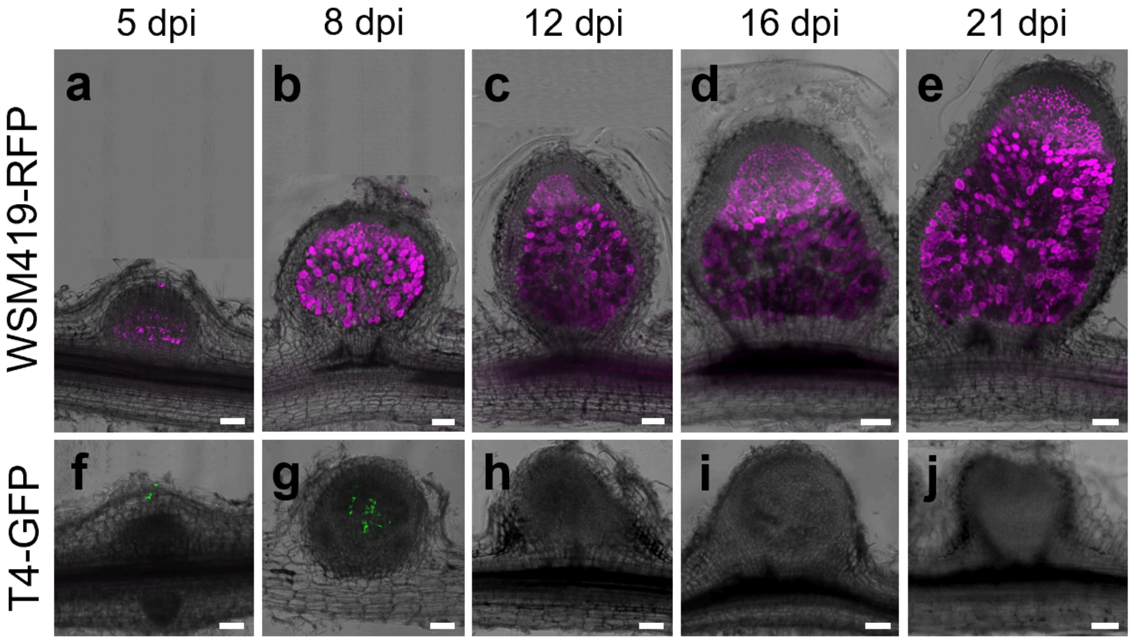

Supplementary Fig. 9 | T4 bacteroids are not persisting within the nodule. a-j WSM419-RFP and T4-GFP bacteroids localization during nodulation. **a-e** From 5 to 21 dpi, WSM419-RFP-derived nodules are densely filled with WSM419-RFP bacteroids (violet signal). **f-g** From 5 to 8 dpi, T4-GFP nodules are poorly filled with alive T4-GFP bacteroids (green signal). **h-j** From 12 to 21 dpi, T4-GFP nodules show barely visible to no GFP signal. (Scale bars **a-j**, 100 μm).

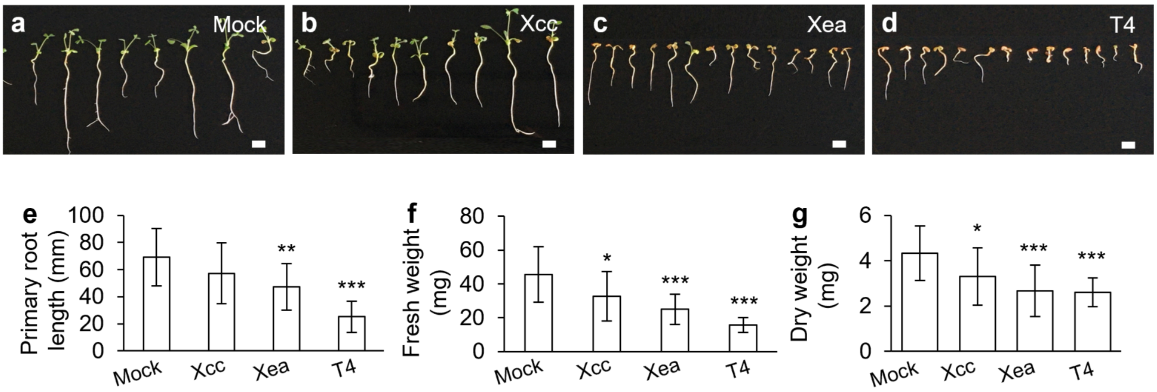

Supplementary Fig. 10 | Comparison of the T4 virulence relative to *Xanthomonas* strains. Comparison of the virulence of the T4 relative to the virulence of *Xanthomonas campestris pv. campestris* (*Xcc*) and *Xanthomonas euvesicatoria pv. alfalfae (Xea)*. **a-d** Representative pictures of mock- **(a)**, Xcc- **(b)**, Xea- **(c)** and T4- **(d)** infected plants at 21 dpi. (Scale bars, 1 cm). **e-g** Quantification of the virulence of *Xcc*, *Xea* and T4 relative to mock based on primary root length **(e)**, plant fresh weight **(f)** and plant dry weight **(g)** at 21 dpi. Data represent means ± SD. (For mock, *Xcc*, *Xea*, T4, n = 13, 24, 24, 24). Asterisks indicate significant differences relative to mock (*, $P \leq 0.05$; **, $P \leq 0.01$; ***, $P \leq 0.001$; Student's *t*-test).

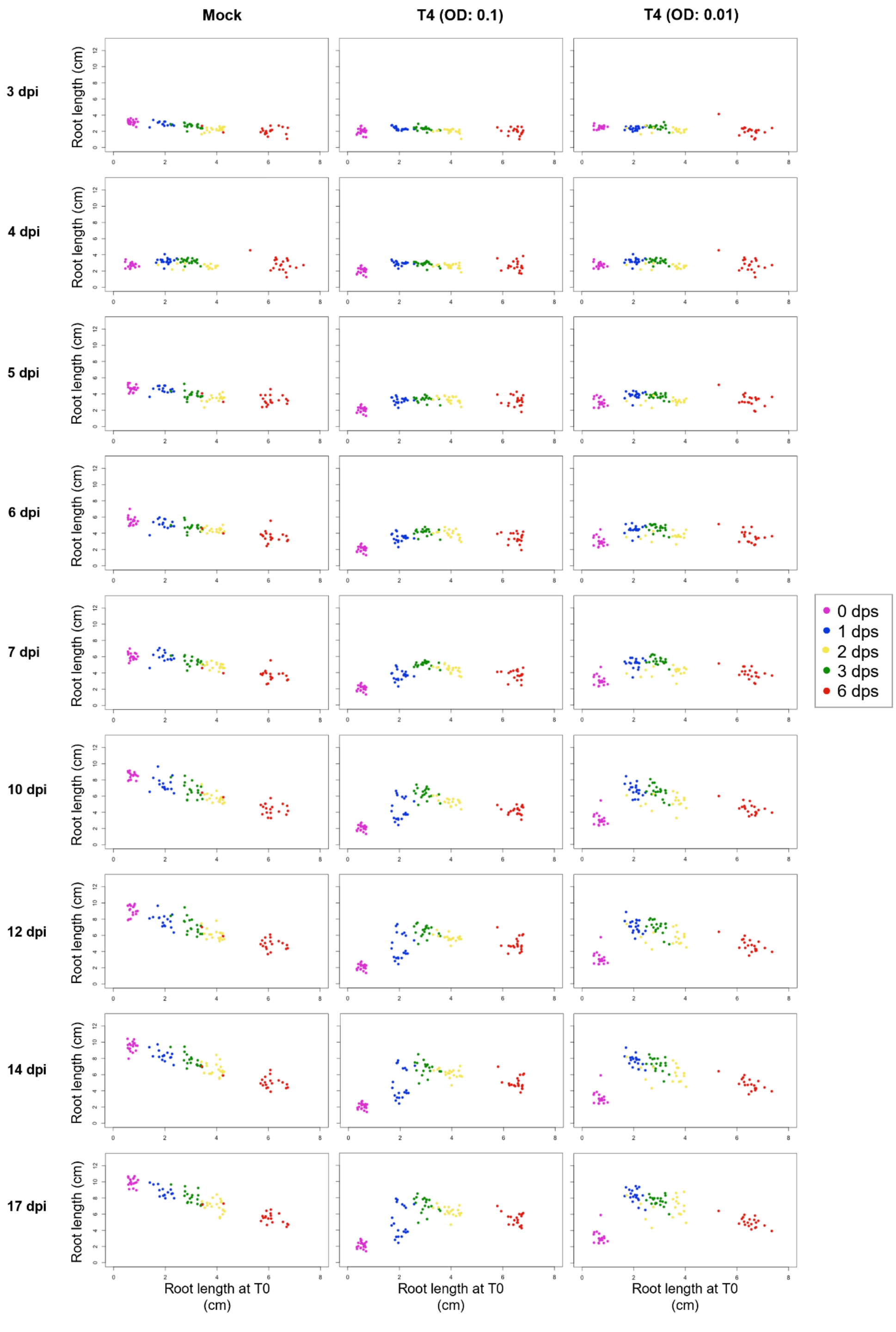

Supplementary Fig. 11 | Susceptible-to-resistant shift of *M. truncatula* A17 in response to T4 inoculation. Exhaustive graphical representation of A17 root length measurements along a 17-day-kinetics (3, 4, 5, 6, 7, 10, 12, 14 and 17 dpi) and with five different timing of T4 inoculation post stratification (0, 1, 2, 3, and 6 dps). Two densities of T4 inoculum have been tested, OD: 0.1 and OD: 0.01). Data represent primary root lengths at t_X minus corresponding primary root lengths at t_0. Experiments consist of three biological replicates (n = 60).

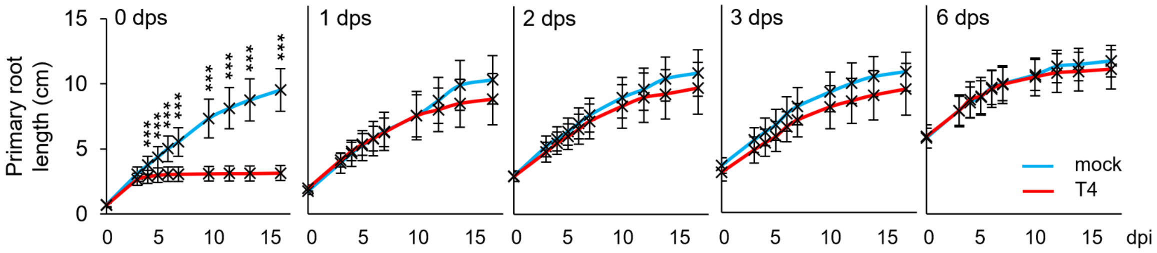

Supplementary Fig. 12 | The bacterial density of the inoculum influences the virulence of strain T4. Timing of the susceptible-to-resistant shift of *M. truncatula*. *M. truncatula* plants were inoculated at 0, 1, 2, 3 and 6 days post stratification (dps) with a T4 suspension adjusted at OD_{600nm}: 0.01 or with mock. The virulence of the T4 was quantified through measuring the primary root length of T4-inoculated plants (Red curves) compared to mock-inoculated plants (Blue curves) along an infection kinetic from zero to 17 dpi. The infection of *M. truncatula* with a T4 inoculum of OD_{600nm}: 0.01 shifts by two days earlier the timing of the susceptible-to-resistant shift of *M. truncatula* in comparison to OD_{600nm}: 0.1 **(Fig. 4)**. Data represent means ± SEM of three biological replicates (n = 60). Asterisks indicate significant differences compared to the mock (***, $P \leq 0.0001$; Student's *t*-test).

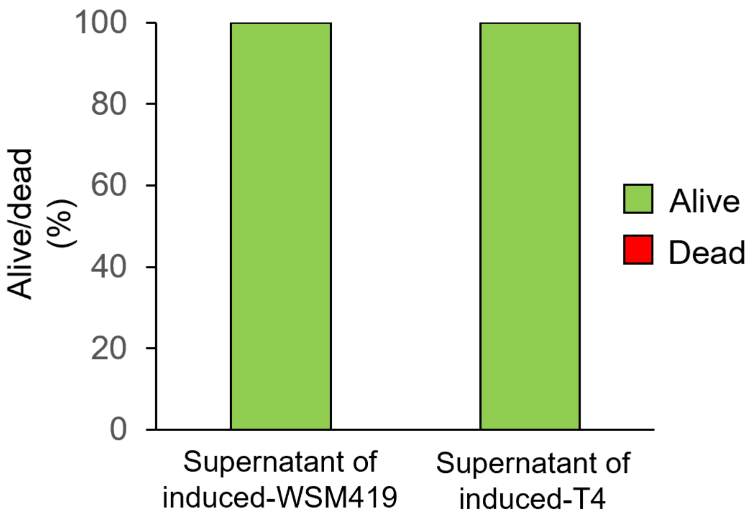

Supplementary Fig. 13 | T4 supernatant alone does not affect plant viability. Inoculation of A17 seedlings with cell-free spent medium of A17 seedling-induced T4 liquid culture does not trigger plant death (For WSM419 and T4, n = 40 and 36 plants, respectively).

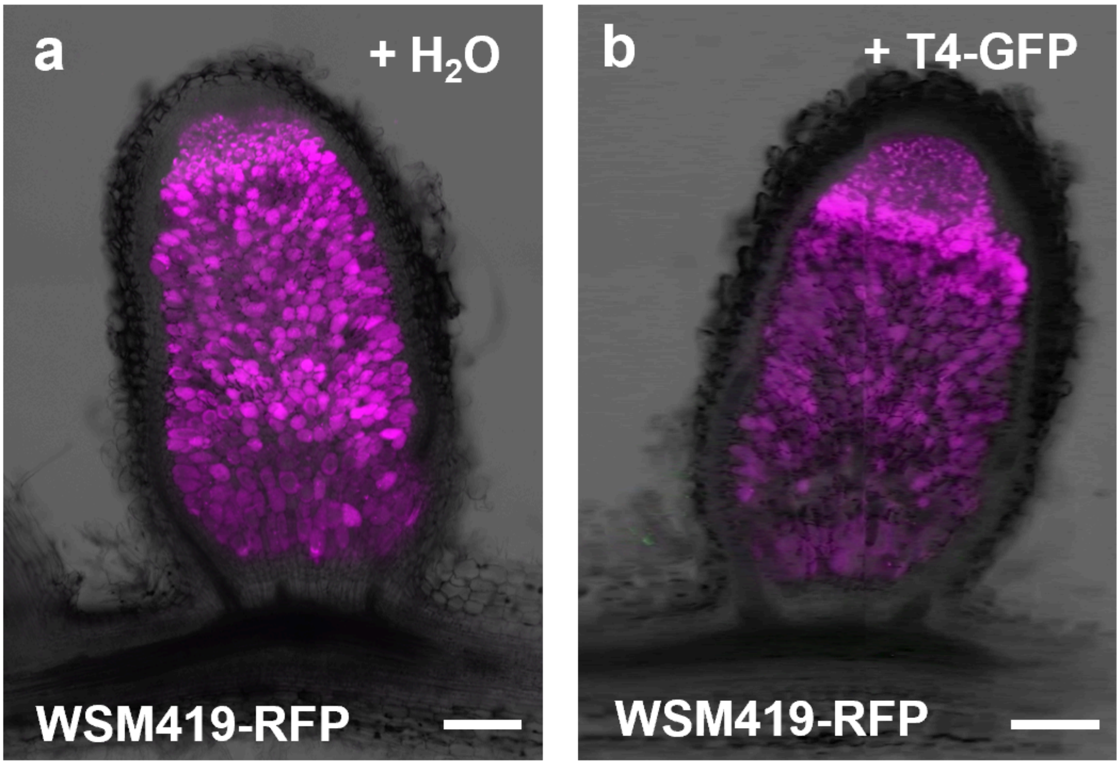

Supplementary Fig. 14 | T4 did not infect established nodules. T4-GFP does not infect 8-dpi WSM419-RFP mature nodules. **a** and **b** Longitudinal section of 21-dpi WSM419-RFP (violet signal) nodules inoculated with H_2O (**a**) or T4-GFP (green signal, **b**) when nodules were eight dpi. WSM419-RFP nodules are strictly occupied by WSM419-RFP bacteroids. T4 were not observed within nodules. (Scale bars, 200µm).

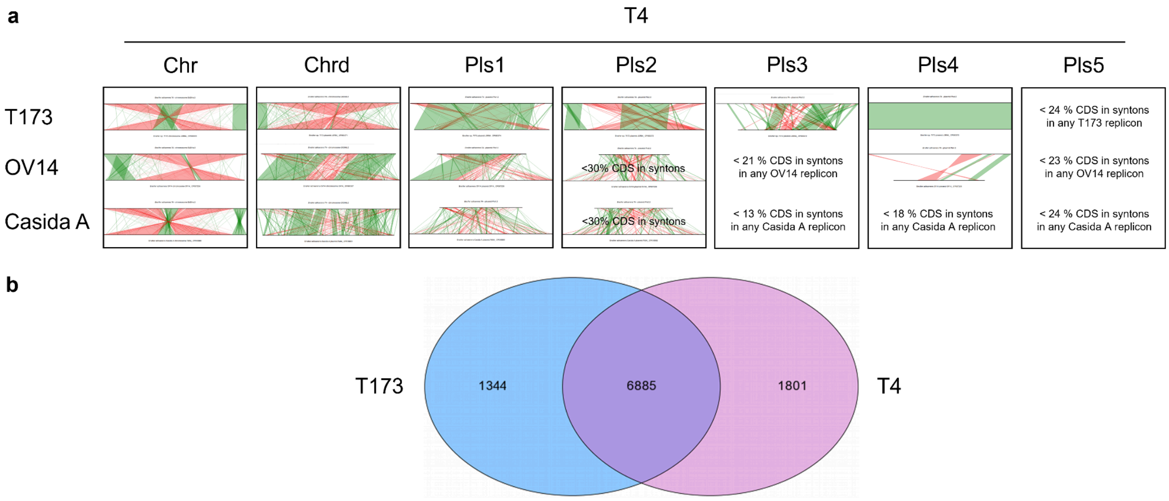

Supplementary Fig. 15 | Comparison of T4 and T173 genomic contents. a Line plots representing overviews of synteny groups between T4 and T173 replicons. Syntenies are also shown for *E. adhaerens* OV14 and *E. adhaerens* Casida A as references. Indicated syntons were defined as ≥ three genes. Syntenic regions are shown in green, inversions around the origin of replication are shown in red. **b** Venn diagram illustrating T4 and T173 discrepancies and similarities at the genome level. The number of specific and common CDS families between T173 and T4 is indicated. 80% amino acid identity and 80% alignment coverage were used as thresholds. Chr, chromosome; chrd, chromid; Psl, Plasmid.

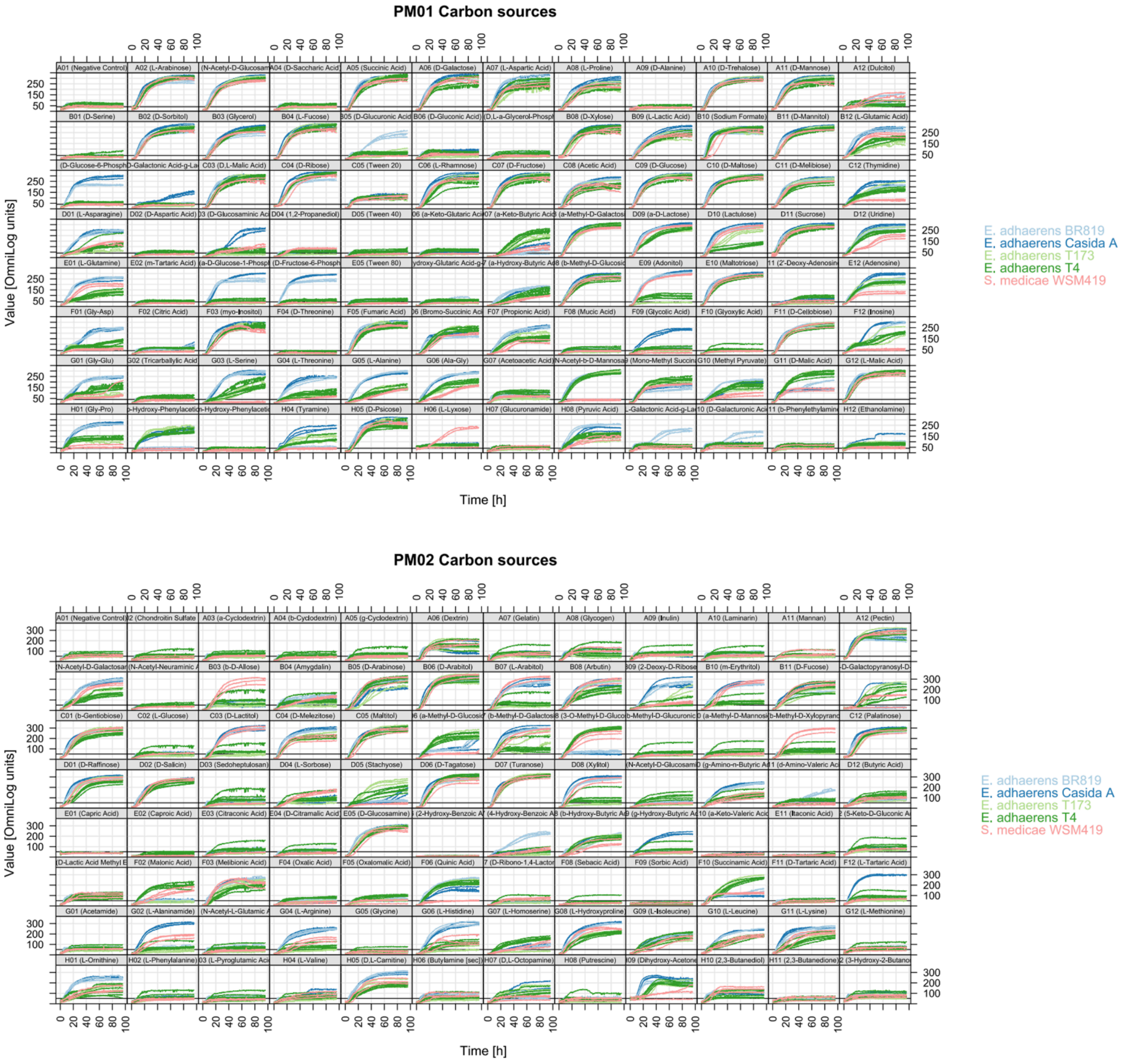

Supplementary Fig. 16 | Biolog phenotype microarray charts for PM01 and PM02 carbon usage plates. Graphical display of the phenotype microarray raw data for each well of the PM01 and PM02 plate containing various carbon sources. The curves correspond to the replicates of *E. adhaerens* BR819 in light blue (3 replicates), *E. adhaerens* Casida A dark blue (3 replicates), *E. adhaerens* T173 light green (3 replicates), *E. adhaerens* T4 (6 replicates) and *S. medicae* WSM419 pink (3 replicates).

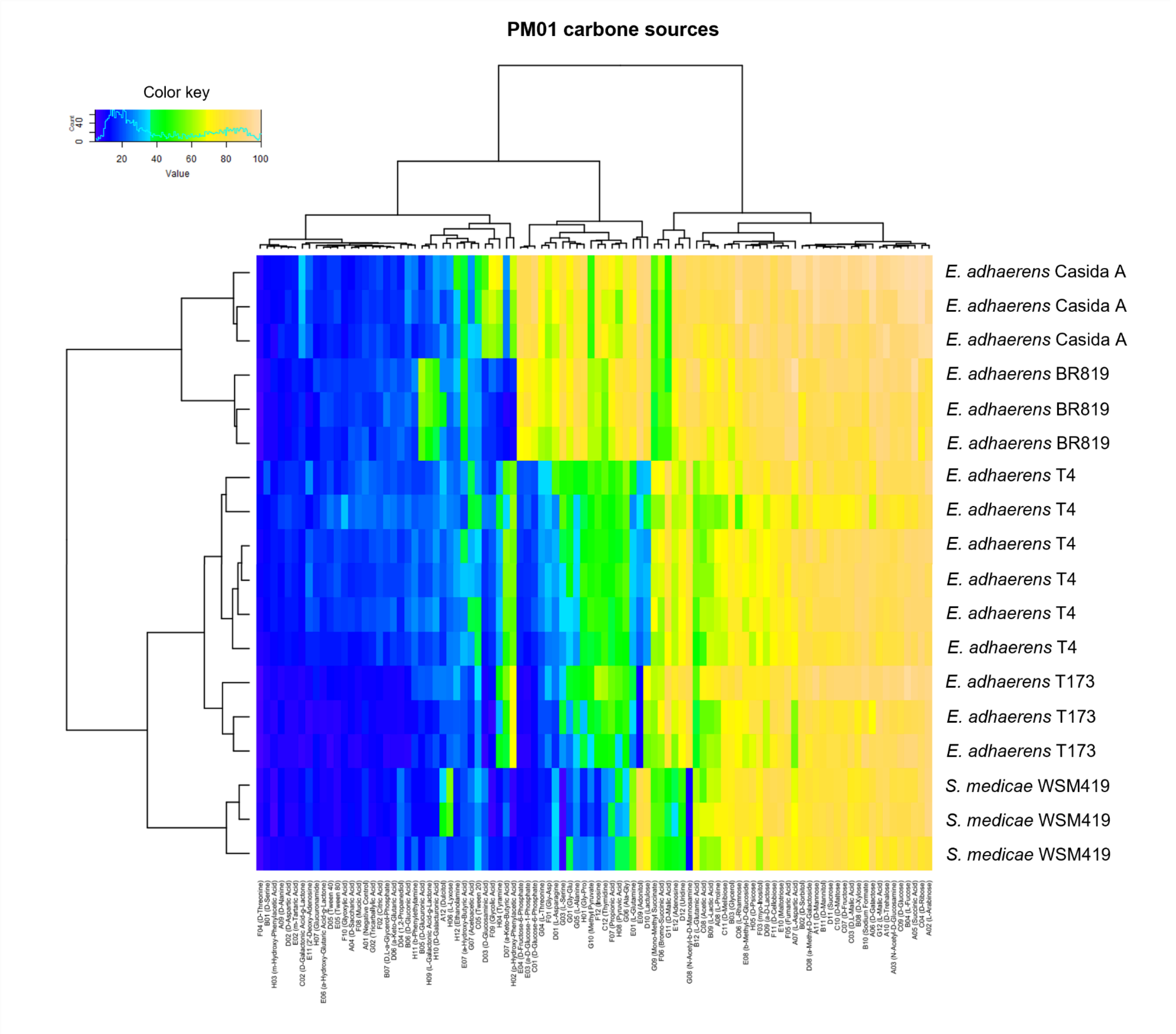

Supplementary Fig. 17 | Biolog phenotype microarray heatmap for the PM01 carbon usage plate. Heat map representation of the PM01 biolog data for all the replicates of strains *E. adhaerens* BR819, *E. adhaerens* Casida A, *E. adhaerens* T173, *E. adhaerens* T4 and *S. medicae* WSM419. The color code corresponds to the area under the curve with increasing value from dark blue (low value), to green (medium value), to yellow (high value) and to beige (very high value).

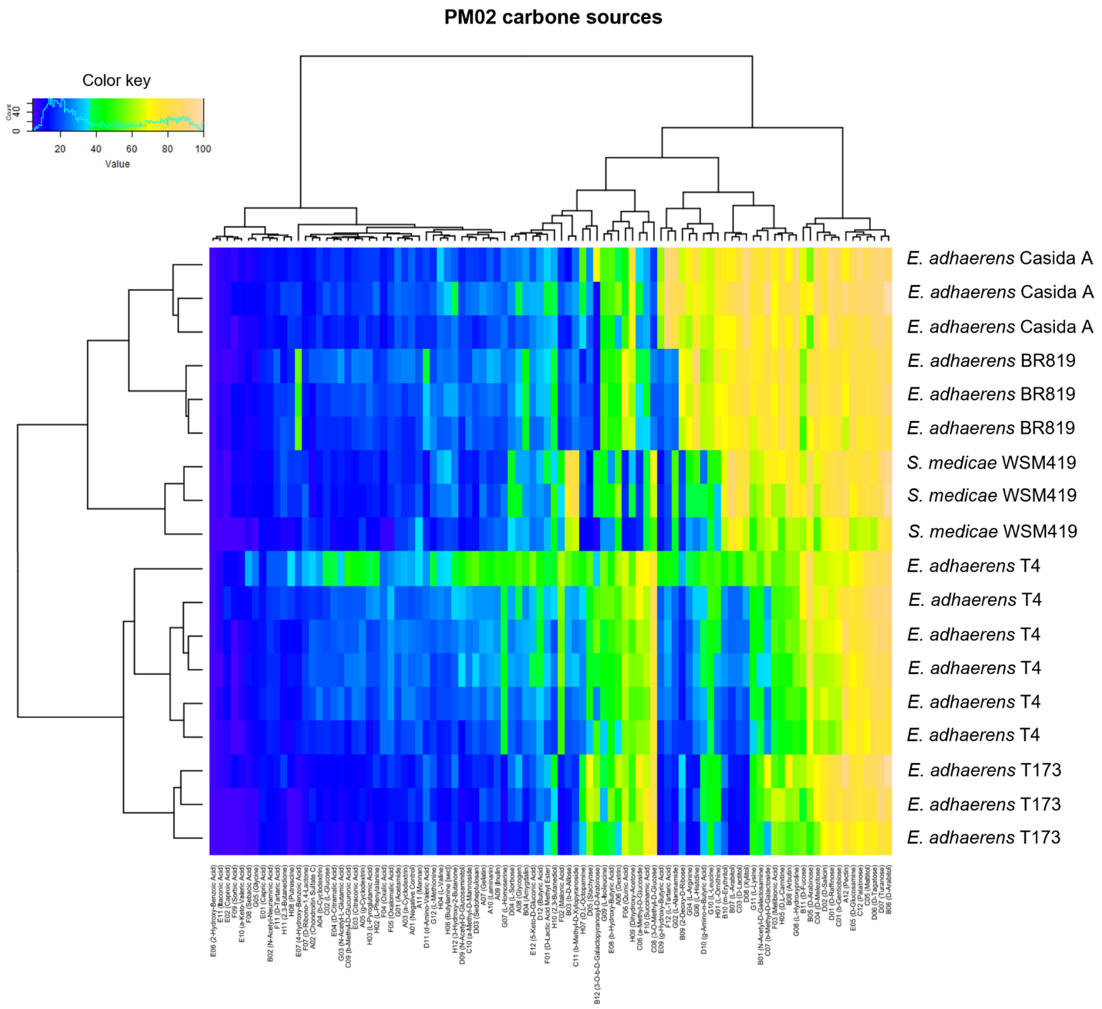

Supplementary Fig. 18 | Biolog phenotype microarray heatmap for the PM02 carbon usage plate. Heat map representation of the PM02 biolog data for all the replicates of strains *E. adhaerens* BR819, *E. adhaerens* Casida A, *E. adhaerens* T173, *E. adhaerens* T4 and *S. medicae* WSM419. The color code corresponds to the area under the curve with increasing value from dark blue (low value), to green (medium value), to yellow (high value) and to beige (very high value).

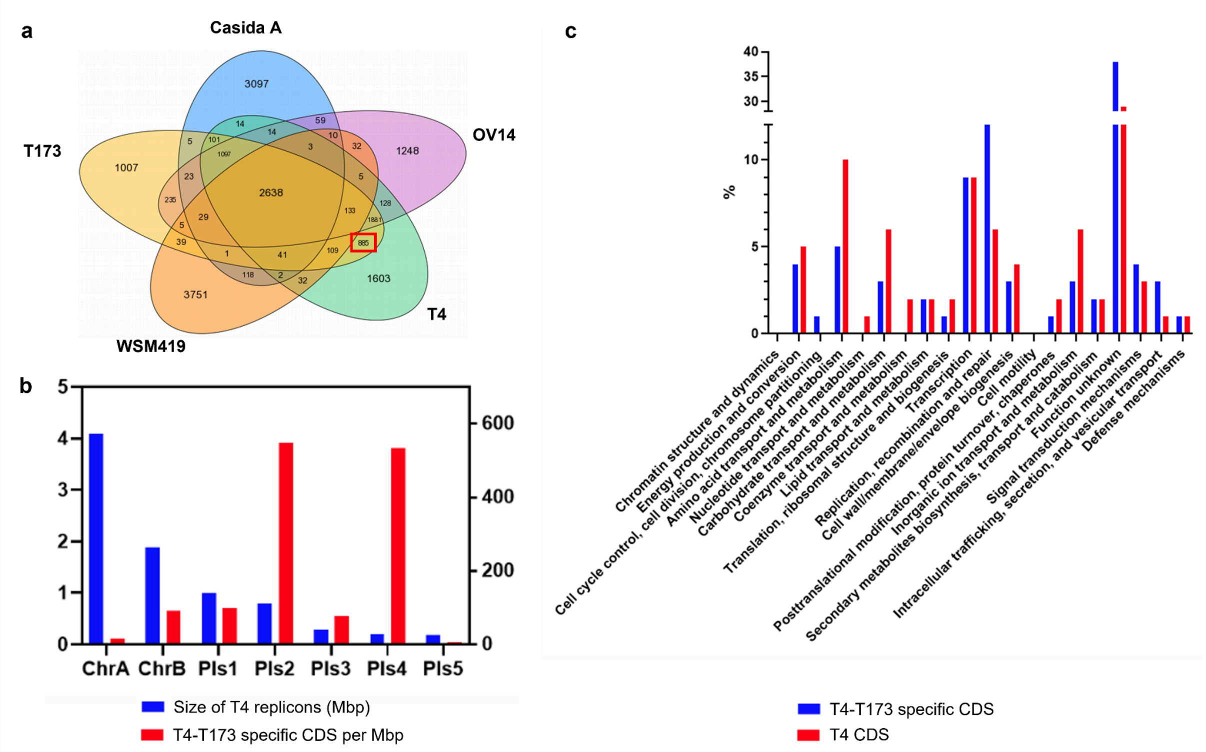

Supplementary Fig. 19 | Comparison of T4, T173, OV14, Casida A and WSM419 genomic contents. a Venn diagram highlighting T4, T173, OV14, Casida A and WSM419 discrepancies and similarities at the genome level. The number of specific and common CDS families is indicated. The red frame highlights CDS families specifically shared between T4 and T173. 80% amino acid identity and 80% alignment coverage were used as thresholds. **b** Distribution of T4-T173 specific CDS families. Red bars show the distribution of 885 T4-T173 specific CDS families along the different T4 replicons (Number of CDS per Mbp, right Y axis). Blue bars show the size of T4 replicons (Mbp, left Y axis). **c** Functional categories of T4-T173 specific CDS families. Red bars represent all T4 CDS families and blue bars represent T4-T173 specific CDS families.

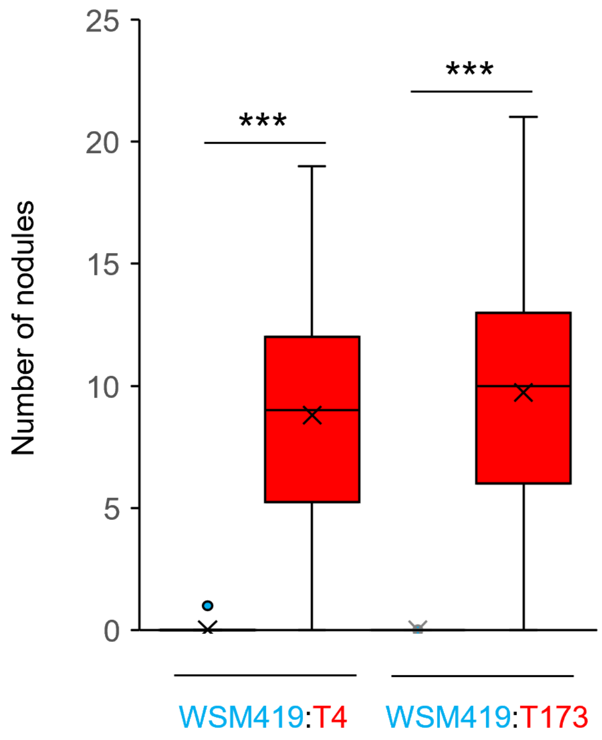

Supplementary Fig. 20 | *E. adhaerens* T173 and T4 competitiveness. As *E. adhaerens* T4 strain, *E. adhaerens* T173 strain dominated nodule formation over the efficient nitrogen-fixing symbionts *S. medicae* WSM419. Strains were co-inoculated following a 1:1 ratio. Each boxplot reflects the analysis of 40 A17 plants. Plants were analyzed at 21 dpi. Asterisks indicate significant differences (***, $P \leq 0.0001$; Student's *t*-test).

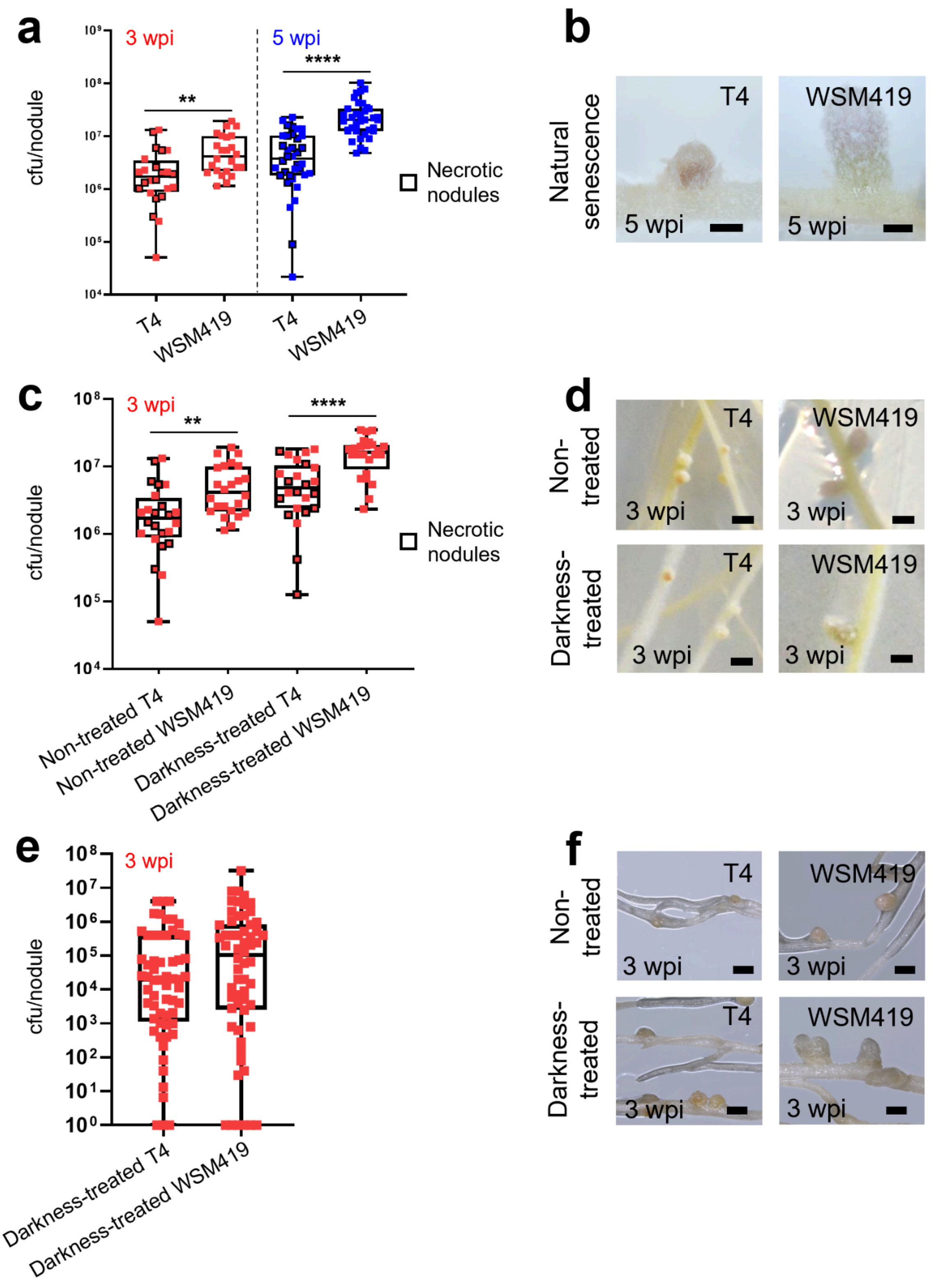

Supplementary Fig. 21 | WSM419 and T4 regrowth from senescent nodules. a cfu counting from single *in vitro*-grown T4 and WSM419 nodules at 3 and 5 weeks post inoculation (wpi). Nodules were surface-sterilized by 70% ethanol for 15 s. Necrotic nodules are highlighted by a black frames. For 3 wpi and 5 wpi, n = 12 and

18 nodules per biological repetition, respectively. The experiment was performed twice. Asterisks indicate significant differences (**, $P \leq 0.001$; ****, $P \leq 0.00001$, Mann & Whitney test). **b** T4 and WSM419 nodules presenting natural senescence phenotypes 5 wpi. (Scale bars, 500 µm). **c** cfu counting from single *in vitro*-grown T4 and WSM419 nodules treated or not by a 3 day-darkness treatment to induce senescence at 3 wpi. Nodules were surface-sterilized by 70% ethanol for 15 s. Necrotic nodules are highlighted by a black frames. n = 12 nodules per biological repetition. The experiment was performed twice. Asterisks indicate significant differences (**, $P \leq 0.001$; ****, $P \leq 0.00001$, Mann & Whitney test). **d** Following the 3 day-darkness treatment WSM419 nodules presented a senescence phenotype (Scale bars, 2 mm). **e** cfu counting from sand-perlite-grown T4 and WSM419 nodules treated by a 3 day-darkness treatment to induce senescence at 3 wpi. Nodules were surface-sterilized by 2 min water/surfactant and 2 min 2.6% sodium hypochlorite. n = 30 nodules per biological repetition. The experiment was performed twice. **f** Following the 3 day-darkness treatment, WSM419 nodules showed a senescence phenotype. (Scale bars, 1 mm).

Supplementary Methods

Phenol staining. Phenol staining was performed as described in the literature[6] with minor modifications. Whole 21-dpi nodules were fixed in sodium cacodylate 0.05 M, pH: 7.2 with glutaraldehyde 1% (v/v) and formaldehyde 4% (v/v) by vacuum infiltration (~500 mm Hg) for 1 h. Samples were washed twice 15 min with sodium cacodylate 0.05 M. 60 µm-thick nodules sections were treated for 30 min with sodium cacodylate 0.05 M, pH 7.2 containing KMnO4 0.02% (w/v) under agitation. Sections were washed using sodium cacodylate 0.05 M, pH 7.2 to remove precipitate for 5 min. Phenolics were stained for 15 min with methylene blue aqueous solution 0.01% (w/v) and cleared with NaOCl (6%) for 2 min and washed with deionized water prior observation.

Nodule phenotyping and quantification. Along the nodulation kinetics, newly formed nodules were annotated at 5, 8, 12, 16 and 21 dpi. Then, six days after the end of the nodulation kinetics, at 27 dpi, nodule numbers were quantified for each timepoint and nodules were classified as a WSM-derived nodules (elongated pink nodules) or as a T4-derived nodules (small white-to-brown nodules).

Cell-free spent medium inoculation. T4 and WSM419 were grown for two days in the presence of germinating A17 seeds. The suspensions were centrifuged for 30 min at 4000 x *g*. The resulting supernatants were double-filtered on 0.22 µm filters. A17 seedlings were then dipped for 1 h in the cell-free spent medium.

Inoculation of established nodules. At eight dpi, A17-WSM419-RFP nodules were spot-infected using 2 µL of T4-GFP suspension (OD_{600nm}: 0,1) supplemented with silwet L-77 0.01% (v/v) as a surfactant. Sterile deionized water supplemented with silwet L-77 0.01% (v/v) was used as control.

Nod factors characterization. Bacterial culture. One liter of bacterial culture was done in V medium (1 g KH_2PO_4, 1 g K_2HPO_4, 0.6 g KNO_3, 0.25 g $MgSO_4$, 0.035 mM $FeCl_3$, 67.1 mg $CaCl_2$, 0.2% Na Succinate, 0.1% Na Glutamate, 500 µg biotin, per 1 L of water), for 24 h, 30°C, 200 rpm. Ten µM of luteolin was added twice at 0 h and 8 h. **NFs Extraction.** NFs were extracted from filtered-culture supernatants by butanol

extraction. The butanol phase was concentrated to dryness, the residue was then dissolved in 0.15 L of water and washed twice with 0.15 L of ethyl acetate. The aqueous fraction was concentrated and lyophilized. The residue was then dissolved in 4 mL acetonitrile 20% and separated in SPE Bond Elut™ C18 SPE Cartridges (Agilent Technologies) in 2 mL of 20%, 50% and 100% acetonitrile. The fractions were dried under nitrogen. Each dry sample of 50% acetonitrile was resuspended in 100 µL of acetonitrile 50%, vortexed, sonicated 3 min, centrifuged 10 min at 13 000 rpm, then transferred to vial and stored to -20°C until analysis. Three independent preparations were done. **Ultra - High - Performance Liquid Chromatography - High - Resolution Mass Spectrometry.** Profiling Ultra - High - Performance Liquid Chromatography - High - Resolution Mass Spectrometry (UHPLC-HRMS) analyses were performed on a Q Exactive Plus quadrupole (Orbitrap) mass spectrometer, equipped with a heated electrospray probe (HESI II) coupled to a U-HPLC Ultimate 3000 RSLC system (Thermo Fisher Scientific, Hemel Hempstead, United Kingdom). Separation was done on a Luna Omega Polar C18 column (150 mm × 2.1 mm i.d., 1.6 µm, Phenomenex, Sartrouville, France) equipped with a guard column. The mobile phase A (MPA) consisted of water with 0.05% formic acid (FA), and the mobile phase B (MPB) consisted of acetonitrile with 0.05% FA. The solvent gradient was 0 min, 2% MPB; 0.5 min, 2% MPB; 10.5 min, 70% MPB; 10.6 min, 98% MPB; 12.6 min, 98% MPA. The flow rate was 0.4 mL/min, the column temperature was set to 40°C, the autosampler temperature was set to 5°C, and the injection volume was fixed to 5 µL. Mass detection was performed in negative ionization (NI) mode at resolution 35,000 power [full width at half-maximum (fwhm) at 400 m/z] for MS1 and 17,500 for MS2 with an automatic gain control (AGC) target of 1 × 106 for full scan MS1 and 1 × 105 for MS2. Ionization spray voltages were set to -3.5 kV, and the capillary temperature was kept at 256°C. The mass scanning range was m/z 100-1500. Each full MS scan was followed by data-dependent acquisition of MS/MS spectra for the six most intense ions using stepped normalized collision energy of 20, 40, and 60 eV. **Data Processing.** UHPLC-HRMS raw data were processed with MS-DIAL version 4.70 for mass signal extraction between 1000 and 1,500 Da from 8 to 13 min[7]. MS1 tolerance was set to 0.01 Da in the centroid mode. The optimized detection threshold was set to 1 × 106. Peaks were aligned with a retention time tolerance of 0.1 min and a mass tolerance of 0.015 Da. Relative quantification was

done for each extract using area, then converted in percentage of the total sum of area of the most abundant detected NFs.

Supplementary References

1 Saitou, N. & Nei, M. The neighbor-joining method: A new method for reconstructing phylogenetic trees. *Molecular Biology and Evolution* **4**:406-425 (1987).

2 Felsenstein, J. Confidence limits on phylogenies: An approach using the bootstrap. *Evolution* **39**:783-791 (1985).

3 Tamura, K., Nei, M. & Kumar, S. Prospects for inferring very large phylogenies by using the neighbor-joining method. *Proceedings of the National Academy of Sciences (USA)* **101**:11030-11035 (2004).

4 Kumar, S., Stecher, G. & Tamura, K. MEGA7: Molecular Evolutionary Genetics Analysis Version 7.0 for Bigger Datasets. *Mol Biol Evol.* **33(7)**:1870-4 (2016).

5 Xiao, T.T., Schilderink, S., Moling, S., Deinum, E.E., Kondorosi, E., Franssen, H., Kulikova, O., Niebel, A. & Bisseling, T. Fate map of Medicago truncatula root nodules fate map of Medicago truncatula root nodules. *Development,* **141**, 3517-3528 (2014).

6 Vasse, J., de Billy, F. & Truchet, G. Abortion of infection during the Rhizobium meliloti-alfalfa symbiotic interaction is accompanied by a hypersensitive reaction. *The Plant Journal* **4(3)**, 555-566 (1993).

7 Tsugawa, H., Cajka, T., Kind, T., Ma, Y., Higgins, B., Ikeda, K., Kanazawa, M., VanderGheynst, J., Fiehn, O. & Arita, M. MS-DIAL: data-independent MS/MS deconvolution for comprehensive metabolome analysis. *Nat Methods.* **12(6)**:523-6 (2015).
