# Supplementary material for: Atypical rhizobia trigger nodulation and pathogenesis on the same legume hosts
Source: Nat Commun. 2024 Oct 26;15:9246. doi: 10.1038/s41467-024-53388-x (PMC11513132; doi:10.1038/s41467-024-53388-x)
Supplement: Supplementary file 3 — Description of Additional Supplementary Files [file 41467_2024_53388_MOESM3_ESM.pdf]

Description of Additional Supplementary Files

Supplementary Data Legends:

Supplementary Data 1. Bacterial strains isolated from M. littoralis R108 nodules and behaviors

Supplementary Data 2. Genome size of Ensifer adhaerens strains

Supplementary Data 3. Ensifer adhaerens general genome features

Supplementary Data 4. Primers used

Supplementary Data 5. matK sequences used to reconstruct the evolutionary history of IRLC species

Supplementary Data 6. Biolog data normalized means PM01 and PM02

Supplementary Data 7. List of T4-T173 specific coding sequences

Supplementary Data 8. List of bacterial strains used in this study
