# Supplementary material for: Atypical rhizobia trigger nodulation and pathogenesis on the same legume hosts
Source: Nat Commun. 2024 Oct 26;15:9246. doi: 10.1038/s41467-024-53388-x (PMC11513132; doi:10.1038/s41467-024-53388-x)
Supplement: Supplementary file 12 — Reporting Summary [file 41467_2024_53388_MOESM12_ESM.pdf]

nature portfolio

Corresponding author(s): Benjamin Gourion
Pascal Ratet

Last updated by author(s): Sep 16, 2024

Reporting Summary

Nature Portfolio wishes to improve the reproducibility of the work that we publish. This form provides structure for consistency and transparency in reporting. For further information on Nature Portfolio policies, see our Editorial Policies and the Editorial Policy Checklist.

Statistics

For all statistical analyses, confirm that the following items are present in the figure legend, table legend, main text, or Methods section.

n/a	Confirmed	
☐	☒	The exact sample size (*n*) for each experimental group/condition, given as a discrete number and unit of measurement
☐	☒	A statement on whether measurements were taken from distinct samples or whether the same sample was measured repeatedly
☐	☒	The statistical test(s) used AND whether they are one- or two-sided<br>*Only common tests should be described solely by name; describe more complex techniques in the Methods section.*
☐	☒	A description of all covariates tested
☒	☐	A description of any assumptions or corrections, such as tests of normality and adjustment for multiple comparisons
☐	☒	A full description of the statistical parameters including central tendency (e.g. means) or other basic estimates (e.g. regression coefficient) AND variation (e.g. standard deviation) or associated estimates of uncertainty (e.g. confidence intervals)
☐	☒	For null hypothesis testing, the test statistic (e.g. *F*, *t*, *r*) with confidence intervals, effect sizes, degrees of freedom and *P* value noted<br>*Give P values as exact values whenever suitable.*
☒	☐	For Bayesian analysis, information on the choice of priors and Markov chain Monte Carlo settings
☒	☐	For hierarchical and complex designs, identification of the appropriate level for tests and full reporting of outcomes
☒	☐	Estimates of effect sizes (e.g. Cohen's *d*, Pearson's *r*), indicating how they were calculated

Our web collection on statistics for biologists contains articles on many of the points above.

Software and code

Policy information about availability of computer code

Data collection	LightCycler 480 software release 1.5.0 SP4<br>cellSens Standard software (OLYMPUS)<br>ZEN2.3 lite software
Data analysis	ImageJ software<br>LinReg PCR: Analysis of Real-Time PCR Data, version 2016.1<br>Flye v.2.9.1 (https://github.com/fenderglass/Flye; Kolmogorov et al., 2019)<br>Gepard (Krumsiek et al., 2007)<br>Ori-Finder 2 (Luo et al., 2014)<br>Microscope platform interface (https://mage.genoscope.cns.fr; Vallenet et al., 2020)<br>MEGA X (Kumar et al., 2018)<br>R package OPM version 1.0.6 (Vaas et al., 2013)<br>MS-DIAL version 4.70 (Tsugawa et al., 2015)

For manuscripts utilizing custom algorithms or software that are central to the research but not yet described in published literature, software must be made available to editors and reviewers. We strongly encourage code deposition in a community repository (e.g. GitHub). See the Nature Portfolio guidelines for submitting code & software for further information.

Data

Policy information about availability of data

All manuscripts must include a data availability statement. This statement should provide the following information, where applicable:

- Accession codes, unique identifiers, or web links for publicly available datasets
- A description of any restrictions on data availability
- For clinical datasets or third party data, please ensure that the statement adheres to our policy

Data availability. All data related to this study are included in the manuscript, in Supplementary information and in Supplementary Data. Source data are provided with this paper. The genomic data of the E. adhaerens T4 strain are accessible on MicroScope - Microbial Genome Annotation & Analysis Platform (https://mage.genoscope.cns.fr) as well as on NCBI (https://www.ncbi.nlm.nih.gov/) under the BioProject ID: PRJNA1066792 [https://www.ncbi.nlm.nih.gov/bioproject/?term=PRJNA1066792]. All genetic materials used in this study are available on request to Pascal Ratet or Benjamin Gourion (pascal.ratet@cnrs.fr; benjamin.gourion@cnrs.fr). The E. adhaerens T4 strain is also available at the CIRM-CFBP French Collection for Plant Associated Bacteria (https://cirm-cfbp.fr/) under the accession number CFBP 9181.

Research involving human participants, their data, or biological material

Policy information about studies with human participants or human data. See also policy information about sex, gender (identity/presentation), and sexual orientation and race, ethnicity and racism.

Reporting on sex and gender	*Use the terms sex (biological attribute) and gender (shaped by social and cultural circumstances) carefully in order to avoid confusing both terms. Indicate if findings apply to only one sex or gender; describe whether sex and gender were considered in study design; whether sex and/or gender was determined based on self-reporting or assigned and methods used. Provide in the source data disaggregated sex and gender data, where this information has been collected, and if consent has been obtained for sharing of individual-level data; provide overall numbers in this Reporting Summary. Please state if this information has not been collected. Report sex- and gender-based analyses where performed, justify reasons for lack of sex- and gender-based analysis.*
Reporting on race, ethnicity, or other socially relevant groupings	*Please specify the socially constructed or socially relevant categorization variable(s) used in your manuscript and explain why they were used. Please note that such variables should not be used as proxies for other socially constructed/relevant variables (for example, race or ethnicity should not be used as a proxy for socioeconomic status). Provide clear definitions of the relevant terms used, how they were provided (by the participants/respondents, the researchers, or third parties), and the method(s) used to classify people into the different categories (e.g. self-report, census or administrative data, social media data, etc.) Please provide details about how you controlled for confounding variables in your analyses.*
Population characteristics	*Describe the covariate-relevant population characteristics of the human research participants (e.g. age, genotypic information, past and current diagnosis and treatment categories). If you filled out the behavioural & social sciences study design questions and have nothing to add here, write "See above."*
Recruitment	*Describe how participants were recruited. Outline any potential self-selection bias or other biases that may be present and how these are likely to impact results.*
Ethics oversight	*Identify the organization(s) that approved the study protocol.*

Note that full information on the approval of the study protocol must also be provided in the manuscript.

Field-specific reporting

Please select the one below that is the best fit for your research. If you are not sure, read the appropriate sections before making your selection.

☒ Life sciences ☐ Behavioural & social sciences ☐ Ecological, evolutionary & environmental sciences

For a reference copy of the document with all sections, see nature.com/documents/nr-reporting-summary-flat.pdf

Life sciences study design

All studies must disclose on these points even when the disclosure is negative.

Sample size	We did not performed sample-size calculation in this work. Sample-size for experiments involving plants usually consists in a minimum 3 independent biological repetitions and is usually based on a "n" that makes statistical analysis effective. We usually favor a minimum of 30 individuals when possible.
Data exclusions	No data were excluded from our ranalysis. All the data were made available in a source data file.
Replication	Experiments were usually replicated 3 times independently, all experiments we did and integrated in this work were reproducible.
Randomization	For every experiments, the position of plants in pots, plants in petri dishes, etc...were randomized as much as possible in order to avoid any

Randomization	position effects.
Blinding	For data collection and analysis, the investigators of this work were not blinded. Blinding was not done because we trust invetigator's integrity.

Reporting for specific materials, systems and methods

We require information from authors about some types of materials, experimental systems and methods used in many studies. Here, indicate whether each material, system or method listed is relevant to your study. If you are not sure if a list item applies to your research, read the appropriate section before selecting a response.

Materials & experimental systems

n/a	Involved in the study
☒	☐ Antibodies
☒	☐ Eukaryotic cell lines
☒	☐ Palaeontology and archaeology
☒	☐ Animals and other organisms
☒	☐ Clinical data
☒	☐ Dual use research of concern
☐	☒ Plants

Methods

n/a	Involved in the study
☒	☐ ChIP-seq
☒	☐ Flow cytometry
☒	☐ MRI-based neuroimaging

Plants

Seed stocks

Medicago truncatula ssp. truncatula ecotype Jemalong A17 were provided by Centre INRAE Nouvelle Aquitaine, Poitier, France.
Pisum sativum var. caméor were provided by Richard Thompson (Agroecologie INRAE, Dijon, France).
Melilotus albus, Melilotus officinalis, Trifolium pratense, Trifolium repens, Trifolium subterraneum, Medicago polymorpha ciliaris, Vicia hirsuta, Lens culinaris and M. litoralis R108 were produced in the team Ratet (IPS2).
A17 proENOD11::gusA and A17 Mtdnf1-1 were obtained from corresponding authors of Journet et al. 2020 and Van de Velde, W. et al 2010 respectively. Medicago sativa (WL903, G969, Salina, Oleron and Super GRI8) and Medicago truncatula (Parragio, F83005.5, Ghor) were provided by Prof. Gruber (IPS2). Trigonella calliceras, Glycine max, Lotus japonicus Gifu, Sesbania rostrata, Astragalus sp., Galega orientalis, Galega officinalis were provided by the Mergaert group I2BC.

Novel plant genotypes

Describe the methods by which all novel plant genotypes were produced. This includes those generated by transgenic approaches, gene editing, chemical/radiation-based mutagenesis and hybridization. For transgenic lines, describe the transformation method, the number of independent lines analyzed and the generation upon which experiments were performed. For gene-edited lines, describe the editor used, the endogenous sequence targeted for editing, the targeting guide RNA sequence (if applicable) and how the editor was applied.

Authentication

Describe any authentication procedures for each seed stock used or novel genotype generated. Describe any experiments used to assess the effect of a mutation and, where applicable, how potential secondary effects (e.g. second site T-DNA insertions, mosiacism, off-target gene editing) were examined.
